# Long-range transport of mutagens and other air pollutants from mainland East Asia to western Japan

**DOI:** 10.1186/s41021-015-0025-5

**Published:** 2015-12-08

**Authors:** Souleymane Coulibaly, Hiroki Minami, Maho Abe, Tomohiro Hasei, Tadashi Oro, Kunihiro Funasaka, Daichi Asakawa, Masanari Watanabe, Naoko Honda, Keiji Wakabayashi, Tetsushi Watanabe

**Affiliations:** Department of Public Health, Kyoto Pharmaceutical University, 1 Misasagi-Shichonocho, Yamashinaku, Kyoto, 607-8412 Japan; Tottori Prefectural Institute of Public Health and Environmental Science, 526-1 Minamidani, Yurihamacho, Tottori, 682-0704 Japan; Osaka City Institute of Public Health and Environmental Sciences, 8-34 Tojocho, Tennojiku, Osaka, 543-0026 Japan; Department of Respiratory Medicine and Rheumatology, Tottori University Faculty of Medicine, 36-1 Nishimachi, Yonago, 683-8504 Tottori Japan; Department of Food and Nutrition, Faculty of Human Health, Sonoda Women’s University, 7-29-1 Minamitsukaguchicho, Amagasaki, 661-8520 Hyogo Japan; Graduate Division of Nutritional and Environmental Sciences, University of Shizuoka, 52-1 Yada, Surugaku, Shizuoka, 422-8526 Japan

**Keywords:** Transboundary air pollution, Total suspended particles, Asian dust, Ames test, Polycyclic aromatic hydrocarbon

## Abstract

**Introduction:**

Asian dust events, transport of dust particles from arid and semi-arid areas in China and Mongolia to the east by prevailing westerlies, are often observed in Japan in spring. In recent decades, consumption of fossil fuels has markedly increased in mainland East Asia with rapid economic growth, and severe air pollution has occurred. A part of air pollutants including mutagens, such as polycyclic aromatic hydrocarbons (PAHs), generated in mainland East Asia are thought to be transported to Japan by the prevailing westerlies, like Asian dust, and winter monsoon. The objective of this study was to clarify the long-range transport of mutagens and other air pollutants in East Asia. Thus, we collected total suspended particles (TSP) at a rural town in western Japan, namely, Yurihama in Tottori Prefecture, for 1 year (June 2012–May 2013), and investigated their chemical constituents and mutagenicity.

**Results:**

Many TSP collected from January to March showed high mutagenicity toward *Salmonella typhimurium* YG1024 with and without S9 mix, and high levels of lead (Pb) and sulfate ions (SO_4_^2−^), which are indicators of transboundary air pollutions from mainland East Asia, were detected in those TSP. A large amount of iron, which is an indicator of sand, was found in highly mutagenic TSP collected in March, but not in TSP collected in January and February. High levels of PAHs were detected in highly mutagenic TSP collected from January to March. The ratios of the concentration of fluoranthene to those of fluoranthene and pyrene suggested that the main source of PAHs in TSP collected in winter and spring was coal and biomass combustion. Backward trajectories of air masses on days when high levels of mutagenicity were found indicated that these air masses had traveled from eastern or northern China to Yurihama.

**Conclusions:**

These results suggest that high levels of mutagens were transported from mainland East Asia to western Japan, and this transportation accompanied Asian dust in March, but not in January and February.

## Introduction

An Asian dust event is a meteorological phenomenon in which dust particles originating in arid and semi-arid areas in western China and Mongolia, such as the Gobi Desert and the Loess Plateau, are transported to the east by prevailing westerlies [1]. The Japan Meteorological Agency (JMA) has assessed Asian dust events in terms of visibility at 60 meteorological observatories across Japan, and has reported that Asian dust events were mainly observed there in spring (March–May) [2]. Moreover, the Light Detection and Ranging (LIDAR) system has been used to measure Asian dust at 12 sites in Japan [3]. The dust extinction coefficient based on LIDAR measurement can be used to estimate the amount of non-spherical dust particles, such as Asian dust. Ueda et al. [4] determined moderate Asian dust days (0.066/km < dust extinction coefficient ≤ 0.105/km) and heavy Asian dust days (0.105/km < dust extinction coefficient) using LIDAR.

In addition to the prevailing westerlies in spring, a winter monsoon blows from mainland East Asia to Japan in winter (December–February). In China, atmospheric concentrations of fine particulate matter (PM_2.5_, airborne particles with an aerodynamic diameter of less than or equal to 2.5 μm) were reported to be high in winter [5]. Winter heating was identified as a major contributor to the severe pollution, and coal combustion was revealed to be a main source [5]. In 2013, the International Agency for Research on Cancer (IARC) reported that outdoor pollution and particulate matter are carcinogenic to humans (Group 1) [6]. Mutagenic/carcinogenic substances, such as polycyclic aromatic hydrocarbons (PAHs), are formed by the incomplete combustion of organic matter such as fossil fuels [7] and these substances were mainly detected in fine particles [8, 9]. Therefore, long-range transport of anthropogenic air pollutants including mutagens may occur in East Asia by the winter monsoon and the prevailing westerlies in winter and spring, respectively. However, there are few reports on transboundary air pollution with mutagens in East Asia.

The objective of this study was to clarify the long-range transport of mutagens in East Asia; for this, we measured air pollutants at Yurihama in Tottori Prefecture, Japan, from June 2012 to May 2013. Yurihama is a rural town located on the west coast of Japan, where there are no major air pollution sources. In this study, air pollution was frequently examined in winter and spring by quantifying total suspended particles (TSP), iron (Fe), lead (Pb), sulfate ions (SO_4_^2−^), nitrate ions (NO_3_^−^), polycyclic aromatic hydrocarbons (PAHs), and bacterial mutagenicity. Fe is a main constituent of the earth’s crust and is an indicator of the amount of sand in the atmosphere [10]. Pb is a minor constituent of the crust and is emitted into the atmosphere by the combustion of coal, and refuse incineration [11]. Anthropogenic emission of sulfur oxides and nitrogen oxides is caused by the combustion of fossil fuels, such as coal and petroleum. The increases of atmospheric Pb and SO_4_^2−^ in Japan suggest air pollution by the long-range transport from mainland East Asia [12]. PAHs are produced by the imperfect combustion of organic matter, such as fossil fuels and biomass [7]. In this study, ten PAHs classified as priority pollutants by the United States Environmental Protection Agency were analyzed to estimate the amounts of PAHs in TSP. The transport routes of air masses were estimated by backward trajectory analysis. We compared our results with the occurrences of Asian dust events found by observations conducted by JMA and the National Institute for Environmental Studies (NIES) using visibility and LIDAR, respectively.

## Materials and methods

### Reagents

Benzo[*b*]fluoranthene (BbF, CAS 205-99-2), benzo[*k*]fluoranthene (BkF, CAS 207-08-9), benzo[*a*]pyrene (BaP, CAS 50-32-8), indeno[1,2,3-*cd*]pyrene (IcdP, CAS 193-39-5), nitric acid (HNO_3_, CAS 7697-37-2), hydrochloric acid (HCl, CAS 7647-01-0), hydrofluoric acid (HF, CAS 7664-39-3), and perchloric acid (HClO_4_, CAS 7601-90-3) were purchased from Wako Pure Chemical Industries, Ltd. (Osaka, Japan). Fluoranthene (FR, CAS 206-44-0) and pyrene (PY, CAS 129-00-0) were obtained from Nacalai Tesque Inc. (Kyoto, Japan). Benz[*a*]anthracene (BaA, CAS 56-55-3), dibenz[*a,h*]anthracene (DahA, CAS 53-70-3), 1-nitropyrene (CAS 5522-43-0), and 2-acetylaminofluorene (CAS 53-96-3) were purchased from Tokyo Chemical Industry Co., Ltd. (Tokyo, Japan). Chrysene (CHR, CAS 218-01-9), benzo[*ghi*]perylene (BghiP, CAS 191-24-2), phenobarbital (CAS 50-06-6), and β-naphthoflavone (CAS 6051-87-2) were purchased from Sigma-Aldrich Co. LLC (St. Louis, MO, USA). Quartz filters were obtained from Pall Life Sciences (Port Washington, NY, USA).

### Sampling methodology and sample preparation

**Fig. 1:**
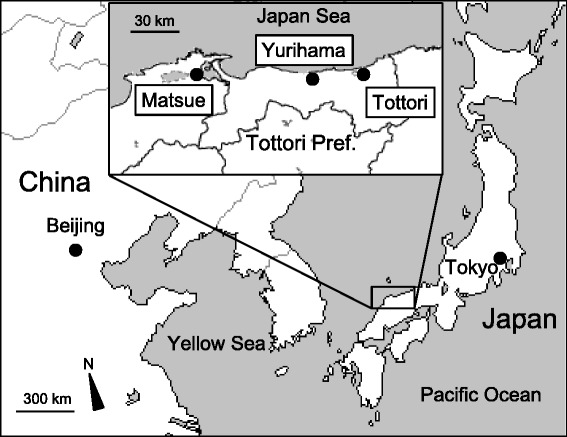
Map of the sampling site and the observation sites of Asian dust event. Yurihama is a sampling site of TSP. Tottori is an observatory site of Asian dust event by JMA [2]. Observation of Asian dust event using LIDAR was performed at Matsue by NIES [3]

TSP were collected on the rooftop of Tottori Prefectural Institute of Health and Environmental Sciences at Yurihama Town (133.88°E, 35.49°N) in Tottori Prefecture. Tottori Prefecture is located in western Japan on the coast of the Japan Sea (Fig. 1). Yurihama is a small town, with a population of about 17,000 in 2013. The sampling site is located at a distance of 1.4 km from the coast and there were no major air pollution sources in the surrounding area. The particulates were collected on quartz filters at a flow rate of 1000 L/min with a high-volume air sampler (HV1000R, Shibata Scientific Technology, Soka, Japan) [8]. Sampling began at about 9 AM (Japan Standard Time, JST) and was completed at about 9 AM the next day. Sampling was performed for 4 days per month from June to November 2012, and from 13 to 19 days per month from December 2012 to May 2013. The total number of sampling days was 118 from June 2012 to May 2013. This sampling period was divided into four seasons, namely, summer (June–August), autumn (September–November), winter (December–February), and spring (March–May).

The filters were weighed before and after sampling after being stored at 50 % relative humidity and 20 °C. To calculate the atmospheric concentration of TSP, the weight of TSP collected on a filter was divided by the volume of the air that had passed through the filter.

### Analysis of metals

Ten percent of each sample filter was cut into small pieces and digested with a mixture of HNO_3_/HCl and then with a mixture of HNO_3_/HF/HClO_4_ [13]. The solution was heated to almost dryness and then cooled to room temperature. After filtration with the addition of distilled water, the sample solution was re-heated and finally adjusted with 0.2 M HNO_3_. Fe and Pb were respectively analyzed by inductively coupled plasma-atomic emission spectrometry (IRIS 1000, Thermo Fisher Scientific, Waltham, MA, USA) and Zeeman electro-thermal atomic absorption spectrometry (Analyst 600, PerkinElmer, Waltham, MA, USA) [14]. When the reference urban atmospheric particles (NIST, SRM1648a) were analyzed by this method, the recovery rates of Fe and Pb were 96 and 83 % (each, *n* = 3), respectively. The limit of quantification (LOQ) of Fe and Pb were as follows: Fe, 99 ng/m^3^; and Pb, 5.1 ng/m^3^.

### Analysis of water-soluble ionic species

Five percent of each sample filter was cut into small pieces and extracted with distilled water by ultra-sonication. A portion of the extract was filtrated using a syringe filter (DISMIC-25CS, Advantec Co., Ltd., Tokyo, Japan). SO_4_^2−^ and NO_3_^−^ were measured using an ion chromatograph (600E/700, Waters Co., Ltd., Milford, MA, USA) with an anion suppressor (ASRS 300, Thermo Fisher Scientific, Waltham, MA, USA) and an anion-exchange column (AS4A-SC, Thermo Fisher Scientific, Waltham, MA, USA) [15]. The LOQ of SO_4_^2−^ and NO_3_^−^ were as follows: SO_4_^2−^, 46 ng/m^3^; and NO_3_^−^, 131 ng/m^3^.

### Analysis of PAHs

Forty percent of each sample filter was cut into small pieces and extracted with methanol by ultra-sonication [8]. After filtration, the extract was evaporated to dryness. Ten PAHs, namely, FR, PY, BaA, CHR, BbF, BkF, BaP, DahA, BghiP, and IcdP, were analyzed by high-performance liquid chromatography using a fluorescence detector (RF-20AXs, Shimadzu Co., Kyoto, Japan). PAHs were separated with a Wakosil-PAHs column (4.6 mm × 250 mm, Wako Pure Chemical Industries, Ltd., Osaka, Japan) and measured with the following excitation (Ex)/emission (Em) wavelengths: FR and PY, 250 nm/420 nm; BaA, CHR, BbF, and BkF, 270 nm/400 nm; BaP, DahA, and BghiP, 296 nm/410 nm; and IcdP, 300 nm/500 nm [8]. The LOQ of PAHs were as follows: FR, 101 fg/m^3^; PY, 165 fg/m^3^; BaA, 14 fg/m^3^; CHR, 98 fg/m^3^; BbF, 127 fg/m^3^; BkF, 63 fg/mm^3^; BaP, 14 fg/m^3^; DahA, 39 fg/m^3^; BghiP, 50 fg/m^3^; and IcdP 112 fg/m^3^.

### Mutagenicity assay

Forty percent of each sample filter was cut into small pieces and extracted with methanol by ultra-sonication [8]. After filtration, the extract was evaporated to dryness. The residues dissolved in dimethyl sulfoxide were assayed using *Salmonella typhimurium* YG1024 in the absence and presence of a mammalian metabolic system, S9 mix [16]. YG1024 was kindly provided by Dr. Nohmi from the National Institute of Health; it is an *O*-acetyltransferase-overproducing derivative of *S. typhimurium* TA98 [17], and is sensitive to the mutagenicity of airborne particles [8, 18]. The S9 mix was prepared with S9 from livers of male Sprague–Dawley rats (SLC Inc., Shizuoka, Japan) treated with phenobarbital and β-naphthoflavone [19]. Positive controls without and with S9 mix were 1-nitropyrene and 2-acetylaminofluorene, respectively. The slope of the dose–response curve obtained with three doses and duplicate plates at each dose was adopted as the mutagenic potency. Samples were judged as positive when they induced twofold increases over the average of spontaneous revertants and showed well-behaved concentration-response patterns.

### Statistical analysis

Statistical analysis (Dunnett’s test, calculation of correlation coefficients, and others) was performed with Microsoft Office Excel 2013. The differences of the diagnostic ratios of PAHs in TSP collected in summer from that in other seasons were analyzed by non-repeated one-way ANOVA followed by Dunnett’s test.

### Backward trajectory analysis

Backward trajectories were estimated with the HYSPLIT model provided by the National Oceanic and Atmospheric Administration (NOAA) of the United States of America [20]. The backward trajectories started at 10 PM (JST), and the height was set at 1500 m. Backward trajectory analysis was performed using model vertical velocity, and the trajectory duration was 72 h.

## Results

### Concentrations of TSP and chemical constituents

Table 1 shows the mean value and standard deviation of the concentration of TSP and the chemical constituents in each season. The highest mean values were obtained in spring for TSP and all chemical constituents, except for total PAHs. We analyzed 10 PAHs to estimate the levels of PAHs in TSP and calculated the sum of the concentrations of these 10 PAHs as total PAHs. The highest mean value for total PAHs was found in winter (1.05 ng/m^3^), and it was close to that of spring (0.92 ng/m^3^). Ten PAHs were detected in almost all TSP. The amounts of a few PAHs, such as DahA, were lower than LOQ in several samples.

**Table 1:** Atmospheric concentrations and mutagenicity of TSP and their chemical constituents

	Summer	Autumn	Winter	Spring
TSP (μg/m^3^)	22.1 ± 12.0	29.3 ± 8.0	22.6 ± 14.3	48.4 ± 37.4
Fe (ng/m^3^)	76.7 ± 66.4	145.7 ± 130.8	126.8 ± 130.5	709.4 ± 844.0
Pb (ng/m^3^)	8.2 ± 13.9	7.3 ± 4.8	9.7 ± 8.5	15.9 ± 15.2
SO_4_^2−^ (μg/m^3^)	5.03 ± 5.50	4.78 ± 2.28	4.36 ± 3.47	7.38 ± 4.88
NO_3_^−^ (μg/m^3^)	0.45 ± 0.22	1.05 ± 0.91	1.39 ± 1.07	2.49 ± 2.70
Total PAHs (ng/m^3^)	0.21 ± 0.16	0.57 ± 0.38	1.05 ± 0.66	0.92 ± 0.73
Mutagenicity (revertant/m^3^)				
Without S9 mix	3.5 ± 1.6	9.7 ± 5.8	19.6 ± 13.7	12.7 ± 12.9
With S9 mix	1.8 ± 1.3	7.2 ± 5.2	17.5 ± 13.4	13.1 ± 18.7

The intervals for collecting TSP were as follows: summer (Jun.–Aug. 2012, *n* = 12), autumn (Sep.–Nov. 2012, *n* = 12),winter (Dec. 2012–Feb. 2013, *n* = 42), and spring (Mar.–May 2013, *n* = 52)

Numbers indicate the mean ± standard deviation

Total PAHs means the sum of the concentrations of 10 PAHs (FR, PY, CHR, BaA, BaP, BbF, BkF, DahA, IcdP, and BghiP)

**Fig. 2:**
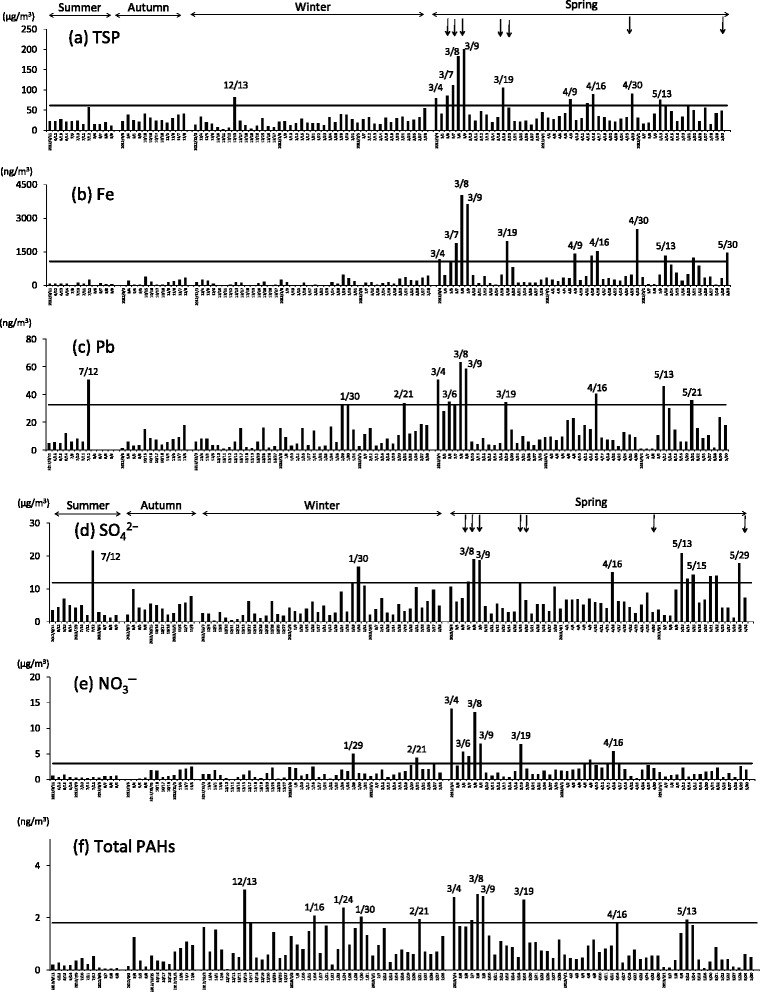
Concentrations of TSP and chemical constituents in TSP collected at Yurihma. Atmospheric concentrations of **a** TSP, **b** Fe, **c** Pb, **d** SO_4_^2−^, **e** NO_3_^−^, and **f** total PAHs were analyzed. The intervals for collecting TSP were as follows: summer (June–August), autumn (September–November), winter (December–February), and spring (March–May). Arrows show dates of Asian dust events registered by the JMA (March 8, 9, 19, and 20) or indicated by LIDAR (March 7, 8, 9, and 19, April 30, and May 30). Horizontal lines show the 90th percentile of TSP (61.0 μg/m^3^), Fe (1061 ng/m^3^), Pb (32.4 ng/m^3^), SO_4_^2−^ (11.8 μg/m^3^), NO_3_^−^ (3.10 μg/m^3^), and total PAHs (1.81 ng/m^3^). Total PAHs means the sum of the concentrations of 10 PAHs (FR, PY, CHR, BaA, BaP, BbF, BkF, DahA, IcdP, and BghiP)

Figure 2 shows the atmospheric concentrations of TSP and chemical constituents in the particles at Yurihama from June 2012 to May 2013. JMA registered Asian dust events on March 8, 9, 19, and 20, 2013, at Tottori, which is 34 km away from the sampling site (Fig. 1). LIDAR measurement was performed at Matsue, which is 73 km away from the sampling site (Fig. 1). On sampling days when dust extinction coefficients were higher than 0.066/km, Asian dust events were considered to have been detected [4], and high dust extinction coefficients (>0.066/km) were found on March 7, 8, 9, and 19, April 30, and May 30, 2013. As shown in Fig. 2, the concentrations of TSP and Fe were remarkably high on March 8 and 9, and high levels of particles and Fe, namely, close to or higher than the 90th percentiles, were found in March and April on common sampling days, such as March 4, 7, 8, 9, and 19, and April 9, 16, and 30. High levels of Pb and SO_4_^2−^ were detected on common sampling days, such as July 12, January 30, March 8, 9, and 19, April 16, and May 13. The atmospheric concentrations of NO_3_^−^ were high on January 29, March 4, 8, 9, and 19, and April 16. As described above, atmospheric concentrations of particles, Fe, Pb, SO_4_^2−^, and NO_3_^−^ were high on many common days, such as March 8, 9, and 19 and April 16. High levels of total PAHs were mainly found from January to March, such as January 16, 24, and 30, February 21, and March 4, 8, 9, and 19. Although high levels of particles and chemical constituents were detected on days when Asian dust events were found by visibility and LIDAR, there were many other sampling days that showed high levels of TSP and chemical constituents.

### Mutagenicity of TSP

The mean value and standard deviation of the mutagenicity of organic extracts of TSP collected at Yurihama in each season are shown in Table 1. The highest mean values were found both without and with S9 mix in winter (without S9 mix, 19.6 revertants/m^3^; with S9 mix, 17.5 revertants/m^3^), and the mean values of mutagenicity without and with S9 mix were the lowest in summer.

**Fig. 3:**
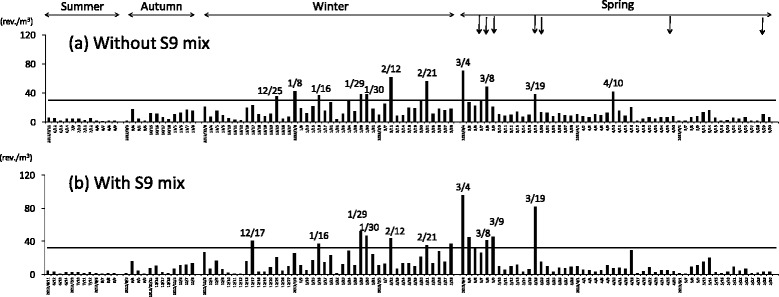
Mutagenicities of TSP collected at Yurihama. Mutagenicity was examined using *Salmonella typhimurium* YG1024 with and without S9 mix. **a** and **b** show mutagenicities of TSP without and with S9 mix, respectively. The intervals for collecting TSP were as follows: summer (June–August), autumn (September–November), winter (December–February), and spring (March–May). Arrows show dates of Asian dust events registered by the JMA (March 8, 9, 19, and 20) or indicated by LIDAR (March 7, 8, 9, and 19, April 30, and May 30). Horizontal lines show the 90th percentile of mutagenicity of organic extracts of TSP in YG1024 without S9 mix (29.9 revertants (rev.)/m^3^) and with S9 mix (32.3 rev./m^3^)

Figure 3 shows the mutagenicities of organic extracts of TSP collected at Yurihama from June 2012 to May 2013 in YG1024 without and with S9 mix. Most of the highly mutagenic samples were collected from January to March, such as January 16, 29, and 30, February 12 and 21, and March 4, 8, and 19. On those days, high mutagenicities were found both without and with S9 mix. Mutagenicities of the whole samples without and with S9 mix were strongly correlated, and the correlation coefficient was 0.840. The highest mutagenicities without and with S9 mix were found on March 4 (70 revertants/m^3^ without S9 mix, 96 revertants/m^3^ with S9 mix).

### Correlations between mutagenicity and concentrations of TSP and their constituents

As shown in Table 1 and Fig. 3, potent mutagenicity was found for samples collected in winter and spring. To analyze the association between the potent mutagenicity and other factors, we carried out statistical analyses for samples collected in winter and spring. Table 2 shows the coefficients of correlations between the mutagenicity and the concentrations of TSP and the constituents in winter and spring. Strong positive correlations, namely, with correlation coefficients greater than 0.7, were obtained for Pb, SO_4_^2−^, NO_3_^−^, and total PAHs without and/or with S9 mix. Moderate positive correlations, namely, with correlation coefficients from 0.5 to 0.7, were found for TSP and Fe without and/or with S9 mix.

**Table 2:** Coefficients of correlation between mutagenicity and concentrations of TSP and their constituents

	Without S9 mix		With S9 mix	
	Winter	Spring	Winter	Spring
TSP	0.458	0.537	0.547	0.600
Fe	0.554	0.490	0.686	0.529
Pb	0.777	0.680	0.853	0.719
SO_4_^2−^	0.663	0.378	0.777	0.407
NO_3_^−^	0.705	0.847	0.670	0.810
Total PAHs	0.657	0.783	0.697	0.853

The intervals for collecting TSP were as follows: winter (Dec. 2012–Feb. 2013, *n* = 42), spring (Mar.–May 2013, *n* = 52). Total PAHs means the sum of the concentrations of 10 PAHs (FR, PY, CHR, BaA, BaP, BbF, BkF, DahA, IcdP, and BghiP)

As shown in Figs. 2 and 3, high levels of contaminants and mutagenicity were found for samples collected in March. Therefore, we carried out statistical analyses for samples collected in March. As shown in Table 3, the concentrations of TSP and Fe were very strongly correlated with that of Pb and SO_4_^2−^, with correlation coefficients greater than 0.9. The concentration of Pb was strongly correlated with the concentrations of other combustion products, namely, SO_4_^2−^, NO_3_^−^, and PAHs. In addition, the mutagenicity of TSP showed strong positive association with the concentrations of Pb, NO_3_^−^, and total PAHs without and with S9 mix.

**Table 3:** Correlation coefficients among the concentrations of TSP and the constituents and the mutagenicity of TSP collected in March

	Concentration						Mutagenicity	
	TSP	Fe	Pb	SO_4_^2−^	NO_3_^−^	Total PAHs	Without S9 mix	With S9 mix
Concentration								
TSP	1.000							
Fe	0.984	1.000						
Pb	0.914	0.901	1.000					
SO_4_^2−^	0.928	0.933	0.891	1.000				
NO_3_^−^	0.755	0.770	0.914	0.780	1.000			
Total PAHs	0.864	0.849	0.941	0.866	0.890	1.000		
Mutagenicity								
Without S9 mix	0.567	0.577	0.804	0.602	0.936	0.830	1.000	
With S9 mix	0.572	0.555	0.780	0.589	0.833	0.864	0.904	1.000

Total PAHs means the sum of the concentrations of 10 PAHs (FR, PY, CHR, BaA, BaP, BbF, BkF, DahA, IcdP, and BghiP)

### Ratio of concentration of FR to that of FR and PY ([FR]/([FR] + [PY]))

**Fig. 4:**
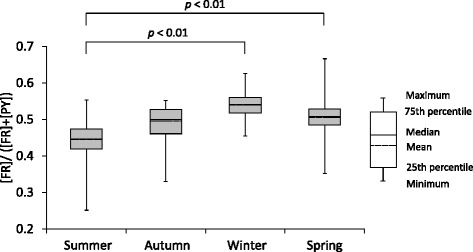
Ratio of concentration of [FR]/([FR] + [PY])) measured at Yurihama. The intervals for collecting TSP were as follows: summer (June–August), autumn (September–November), winter (December–February), and spring (March–May). Statistical analysis was performed on the ratios of [FR]/([FR] + [PY]) by Dunnett’s test

To estimate the source of PAHs, the ratio of the concentration of FR to that of FR and PY ([FR]/([FR] + [PY])) was calculated. Figure 4 shows the [FR]/([FR] + [PY]) ratio for TSP collected at Yurihama in each season. The mean values of [FR]/([FR] + [PY]) ratio measured in winter and spring were 0.54 and 0.50, respectively. The ratios measured in these seasons were significantly higher (*p* < 0.01) than that in summer. On the other hand, the mean value of [FR]/([FR] + [PY]) ratio measured in autumn (0.48) was higher than that in summer (0.44), but the difference of the ratios between the two seasons was not significant.

### Backward trajectory analysis

**Fig. 5:**
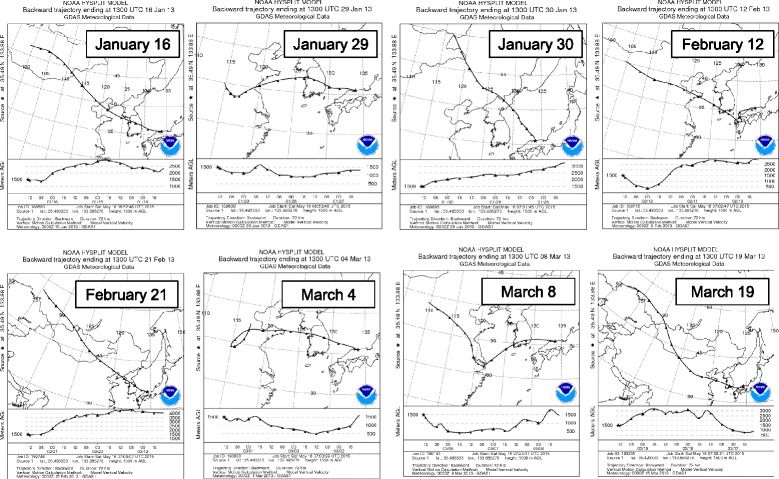
Backward trajectories of air masses from Yurihama. Backward trajectories of air masses for 72 h were calculated using the HYSPLIT model provided by the NOAA of the USA [22]

Figure 5 shows backward trajectories of the air masses on days when high levels of mutagenicity were found, namely, the mutagenic activities were close to or higher than the 90th percentile of the whole sample (Fig. 3). Trajectories from Yurihama on January 29 and March 4 and 8 indicate that air masses had traveled from eastern China. On the other hand, air masses that arrived on January 16 and 30, February 12 and 21, and March 19 were considered to have come from northern China, where the Gobi Desert is located. The backward trajectories of the air masses on February 28 and March 5, when high levels of mutagenicity were found (Fig. 3), were similar to those on January 30 and March 19, and air masses were speculated to have moved from northern China (data not shown). Similarly, the backward trajectories of air masses on March 7 and 9, when high values were found for most of the indicators of air pollution analyzed in this study, resembled that on March 8 (data not shown).

## Discussion

To clarify the effect of the long-range transport of air pollutants from mainland East Asia on the air in western Japan, we collected TSP at Yurihama for 1 year from June 2012 to May 2013, and quantified their chemical constituents and genotoxicity. Yurihama is located in western Japan on the coast of the Japan Sea, and there were no major air pollution sources in the surrounding area. In this study, we analyzed the inorganic and organic substances and mutagenicity. It is well-known that PAHs are generated by imperfect combustion of organic matter, such as fossil fuels [7], and many PAHs show mutagenicity toward *S. typhimurium* YG1024 with S9 mix [21]. High mutagenicity was found for many TSP collected at Yurihama from January to March without and with S9 mix, and the activity level was comparable to those found in medium-sized cities in Japan, such as Sasebo city [8]. The mutagenic activity of TSP was moderately or strongly correlated with the atmospheric concentration of total PAHs and other combustion products, such as NO_3_^−^, both without and with S9 mix (Table 2). Because PAHs are formed by the combustion of organic matter, dominant mutagens in the TSP may be combustion products, and a part of the mutagenicity of TSP with S9 mix may be attributable to PAHs.

In January 2013, a long-lasting episode of severe haze occurred in northern, central, southern, and eastern China [22, 23]. Wang et al. [22] reported that the two most severe episodes occurred during January 9–15 and 25–31 in the Beijing-Tianjin-Hebei area, and monthly averaged daily concentrations of fine particulate matter (PM_2.5_) were very high. Mutagenicity and mutagens, such as PAHs, were mainly detected in fine particles [8]. In the present study, many of the highly mutagenic samples, which were close to or higher than the 90th percentile of the whole sample, were collected at Yurihama in January and February, such as January 29 and 30, and February 21, and high levels of total PAHs were found on those days (Fig. 2). The atmospheric concentrations of Pb and SO_4_^2−^ were also high, but those of TSP and Fe were moderate (Fig. 2). As shown in Fig. 5, the backward trajectories suggest that air masses that arrived on those days had come from northern or eastern China. These results suggest that mutagenic substances were transported from mainland East Asia without a large amount of soil, namely, Asian dust, in January and February.

On the other hand, high levels of TSP and chemical constituents (Fe, Pb, SO_4_^2−^, NO_3_^−^, and PAHs) were found in March, such as on March 4, 7, 8, 9, and 19 (Fig. 2). High mutagenicity was found for TSP collected on those days. Backward trajectories of air masses on those days indicated that the air masses had traveled from mainland East Asia (Fig. 5). High correlation coefficients were found between the concentration of TSP and those of the chemical constituents and mutagenic activity without and with S9 mix (Table 3). JMA registered Asian dust events on March 8, 9, and 19 [2], and average dust extension coefficients higher than 0.066/km, which indicates the occurrence of an Asian dust event [4], were found by LIDAR at Matsue on March 7, 8, 9, and 19 [3]. These results suggest that anthropogenic pollutants including mutagens were transported with Asian dust from mainland East Asia to Yurihama in March.

Several kinds of PAHs ratios were used to estimate the source of PAHs, and the [FR]/([FR] + [PY]) ratio was most commonly used for PAHs in airborne particles [24–26]. To speculate the source of PAHs, we calculated the [FR]/([FR] + [PY]) ratios in TSP collected at Yurihama in this study. Several studies investigated the [FR]/([FR] + [PY]) ratios for many kinds of emitted particles and revealed that the ratios were greater than 0.5 for most coal and wood combustion samples, but smaller than 0.5 for petroleum combustion [24]. Liu et al. [25] measured atmospheric [FR]/([FR] + [PY]) ratios at 46 sites in northern China in winter and reported that the ratios for most samples were greater than 0.5, indicating a predominant influence of coal/biofuel combustion. Similarly, Wang et al. [26] examined [FR]/([FR] + [PY]) ratios in Shanghai in autumn, winter, and spring. They revealed that the ratio in autumn was smaller than 0.5 and those in winter and spring were greater than 0.5; they concluded that wood and biomass burning was the largest source of PAHs contamination in the city. As shown in Fig. 4, the mean values of [FR]/([FR] + [PY]) ratio for TSP collected at Yurihama in winter and spring were greater than 0.5 and both of them were significantly larger than that in summer. These results suggest that high levels of PAHs detected at Yurihama from January to March may be largely affected by coal and biomass combustion, like the atmosphere in northern and eastern China, and PAHs were transported from mainland East Asia.

## Conclusion

Many TSP collected at Yurihama, a rural town in western Japan, from January to March showed high mutagenicity toward *Salmonella typhimurium* YG1024 without and with S9 mix. High levels of Pb and SO_4_^2−^, which are indicators of transboundary air pollutions from mainland East Asia, were detected in the TSP. High levels of iron, which is an indicator of sand, was found in the highly mutagenic TSP collected in March, but not in TSP collected in January and February. High levels of PAHs were detected in the highly mutagenic TSP collected from January to March. The ratio of [FR]/([FR] + [PY]) in the TSP collected in winter and spring suggested that main source of the PAHs was coal and biomass combustion, and that was reported to be major source of air pollutants in northern and eastern China. Backward trajectories of air masses on days when high levels of mutagenicity were found indicated that these air masses had traveled from northern or eastern China to Yurihama. These results suggest that high levels of mutagens were transported from mainland East Asia to western Japan, and this transportation accompanied Asian dust in March, but not in January and February.

### Availability of supporting data

The data sets supporting the results of this article are included within the article.

## Abbreviations

PAHs: Polycyclic aromatic hydrocarbons
TSP: Total suspended particles
Pb: Lead
SO_4_^2−^: Sulfate ion
JMA: Japan Meteorological Agency
LIDAR: Light Detection and Ranging
IARC: International Agency for Research on Cancer
Fe: Iron
NO_3_^−^: Nitrate ion
NIES: National Institute for Environmental Studies
BbF: Benzo[*b*]fluoranthene
BkF: Benzo[*k*]fluoranthene
BaP: Benzo[*a*]pyrene
IcdP: Indeno[1,2,3- *cd*]pyrene
HNO_3_: Nitric acid
HCl: Hydrochloric acid
HF: Hydrofluoric acid
HClO_4_: Perchloric acid
FR: Fluoranthene
PY: Pyrene
BaA: Benz[*a*]anthracene
DahA: Dibenz[*a,h*]anthracene
CHR: Chrysene
BghiP: Benzo[*ghi*]perylene
Ex: Excitation
Em: Emission
NOAA: National Oceanic and Atmospheric Administration
